# Prognostic value of long non-coding RNA TUG1 in various tumors

**DOI:** 10.18632/oncotarget.20025

**Published:** 2017-08-07

**Authors:** Na Li, Ke Shi, Xinmei Kang, Wei Li

**Affiliations:** ^1^ Department of Pathology, The First Affiliated Hospital of Hunan University of Medicine, Huaihua, Hunan Province, People’s Republic of China; ^2^ Department of Geriatrics, Clinical Laboratory, Xiangya Hospital of Central South University, Changsha, Hunan Province, People’s Republic of China

**Keywords:** TUG1, cancer, clinical outcome, prognosis

## Abstract

Taurine up-regulated gene 1 (TUG1) is a long non-coding RNA (lncRNA), has been reported that be dysregulated in various tumors, involved in proliferation and apoptosis in a variety of tumor cells. To detect the clinical significance of TUG1 expression in tumor patients, we carried out current systematic review and meta-analysis investigating its relation with the prognosis and clinicopathological features of cancers. A total of 15 studies comprise 1560 patients were analyzed. The pooled results showed that no significant relationship between high TUG1 expression and overall survival (OS) (HR = 1.28, 95% CI: 0.96–1.69, *P =* 0.091) in various tumors. In the subgroup analysis by cancer type, elevated TUG1 expression was associated with poorer survival in cancer patients with high TUG1 expression subgroup but better survival in patients with low TUG1 expression subgroup. Over-expression of TUG1 associated with significantly unfavorable survival for bladder cancer (HR=2.67, 95% CI: 1.47–4.87, *P =* 0.001). Up-regulation of TUG1 correlated with distant metastasis (DM) (OR = 4.22, 95% CI: 2.66–6.70, *P* < 0.001) and tumor differentiation (OR = 2.45, 95% CI: 1.28–4.70, *P =* 0.007), but failed to show inline to gender (OR = 1.04, 95% CI: 0.77–1.42, *P =* 0.774), age (OR = 0.75, 95% CI: 0.51–1.10, *P =* 0.136), lymph node metastasis (LNM) (OR = 1.45, 95% CI: 0.85–2.50, *P =* 0.177), and TNM stage (OR = 0.55, 95% CI: 0.17–1.81, *P =* 0.326). The overall results suggest lncRNA TUG1 may be a useful prognostic biomarker in cancer patients.

## INTRODUCTION

Taurine up-regulated gene 1 (TUG1) is a newly identified lncRNA, located at chromosome 22q12 with a length of about 7.1kb. It was first reported as a gene up-regulated by taurine in the development of mouse retinal cells [1]. The lncRNA TUG1 also was found that involved in regulating mitochondrial bioenergetics by affected the expression of PGC-1α in diabetic nephropathy [2]. Furthermore, previously study has showed that decreased lncRNA TUG1 expression promoted mouse pancreatic β cells apoptosis and reduced insulin secretion [3]. In addition, up-regulated TUG1 prevent mouse livers from cold induced damage by suppressing cell apoptosis and inflammation [4]. Together, these results indicate that lncRNA TUG1 plays an important role in regulating the development of multiple normal biological processes.

Similarly, numerous studies have reported that TUG1 contribute to proliferation and apoptosis in a variety of tumor cells [5–7]. Increased lncRNA TUG1 expression promotes cell proliferation, metastasis and inhibits cell apoptosis to act as an oncogene in various cancers, such as breast cancer (BRC) [7], colorectal cancer (CRC) [8], ovarian cancer (OC) [9] and small cell lung cancer (SCLC) [10]. However, TUG1 as a tumor suppressor in some other tumors including non-small cell lung cancer (NSCLC) [11, 12], glioma [13], and urothelial carcinoma (UC) [14], up-regulated TUG1 inhibit cell proliferation and reduce tumorigenicity. Therefore, the role of TUG1 in the development and progression of tumours is inconsistent.

Besides, the prognostic value of TUG1 expression in cancer patients also with this same contradiction. Increased TUG1 expression in breast cancer (BRC) [7], bladder cancer (BC) [15], esophageal squamous cell carcinoma (ESCC) [16], muscle-invasive bladder cancer (MIBC) [17], osteosarcoma (OSA) [18], colorectal cancer (CRC) [8], gastric cancer (GC) [19], small cell lung cancer (SCLC) [10] or clear-cell renal cell carcinoma (ccRCC) [20] patients is associated with poor survival. However, in patients with non-small cell lung cancer (NSCLC) [11, 12], urothelial carcinoma (UC) [14] or glioma [13], the increased TUG1 expression is correlated with favorable survival. Thus, we performed present systematic review and meta-analysis to explore the reason for these inconsistent phenomena and investigate the clinical values of TUG1 expression level in various tumors. We mainly discussed the expression of TUG1 associate with prognosis, and metastasis of cancer patients. It aimed to more precisely assess the correlation between TUG1 expression and clinical outcome of human cancers.

## RESULTS

### Study characteristics

**Figure 1:**
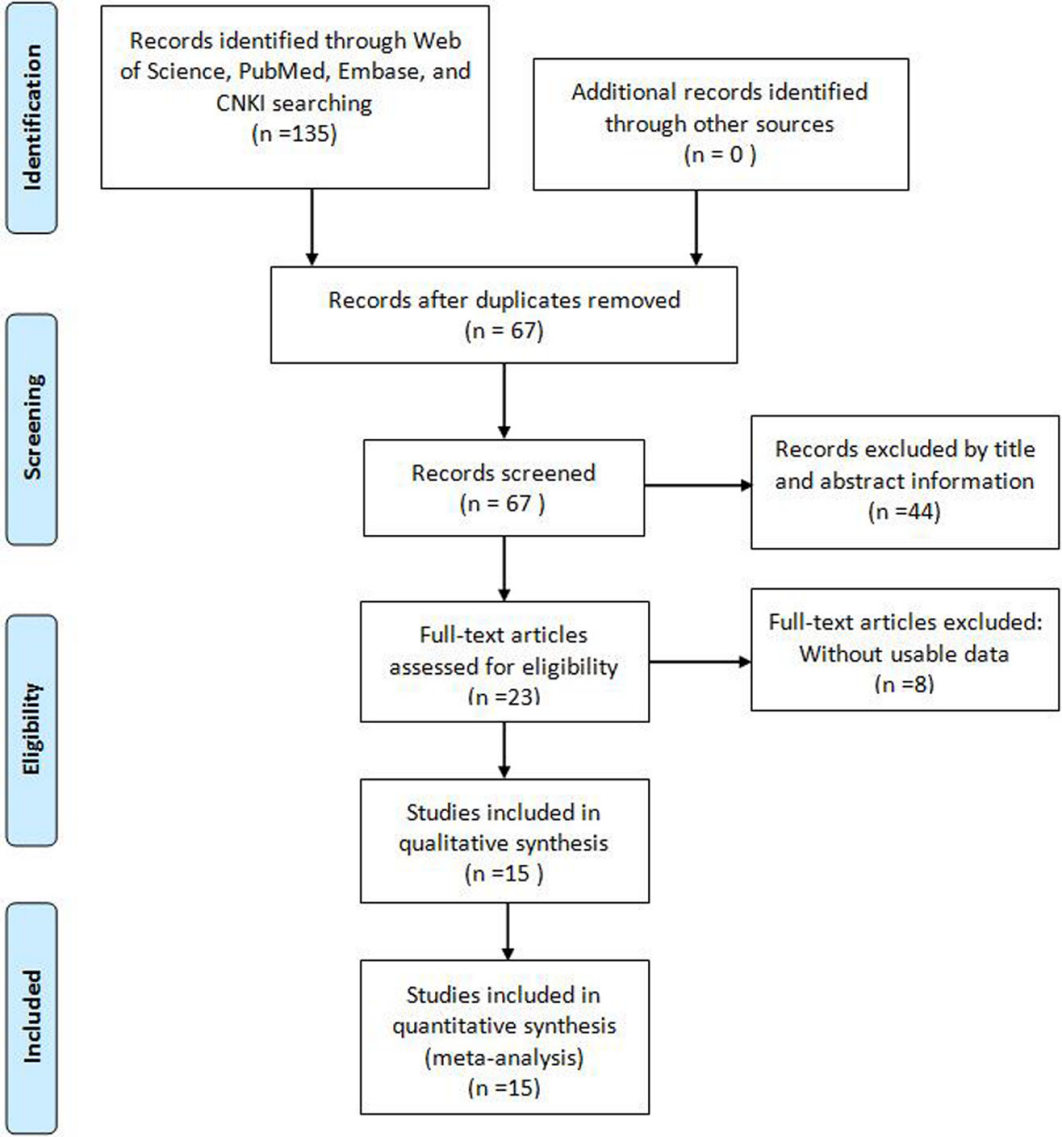
Flow diagram of the literature search and selection

As showed in the flowchart (Figure 1), a total of 135 potentially relevant studies were searched from Embase, PubMed, Web of Science, and China Knowledge Resource Integrated (CNKI) databases. After excluding duplicates, 67 reports were preserved. After reviewing the title and abstracts, 44 records were removed. Subsequently, from the 23 remaining articles 8 were removed due to insufficient data. Finally, a total of 15 studies comprise 1560 patients were included in this meta-analysis [6–20]. Among these cancers derived from 13 tumor types: non-small cell lung cancer [11, 12], bladder cancer [15], muscle-invasive bladder cancer [17], esophageal squamous cell carcinoma [16], ovarian cancer [9], glioma [13], breast cancer [7], osteosarcoma [18], colorectal cancer [8], gastric cancer [19], clear-cell renal cell carcinoma [6, 20], urothelial carcinoma [14], and small cell lung cancer (SCLC) [10]. In all cases, TUG1 expression was detected by qRT-PCR.

### Correlation of TUG1 expression with overall survival

**Figure 2:**
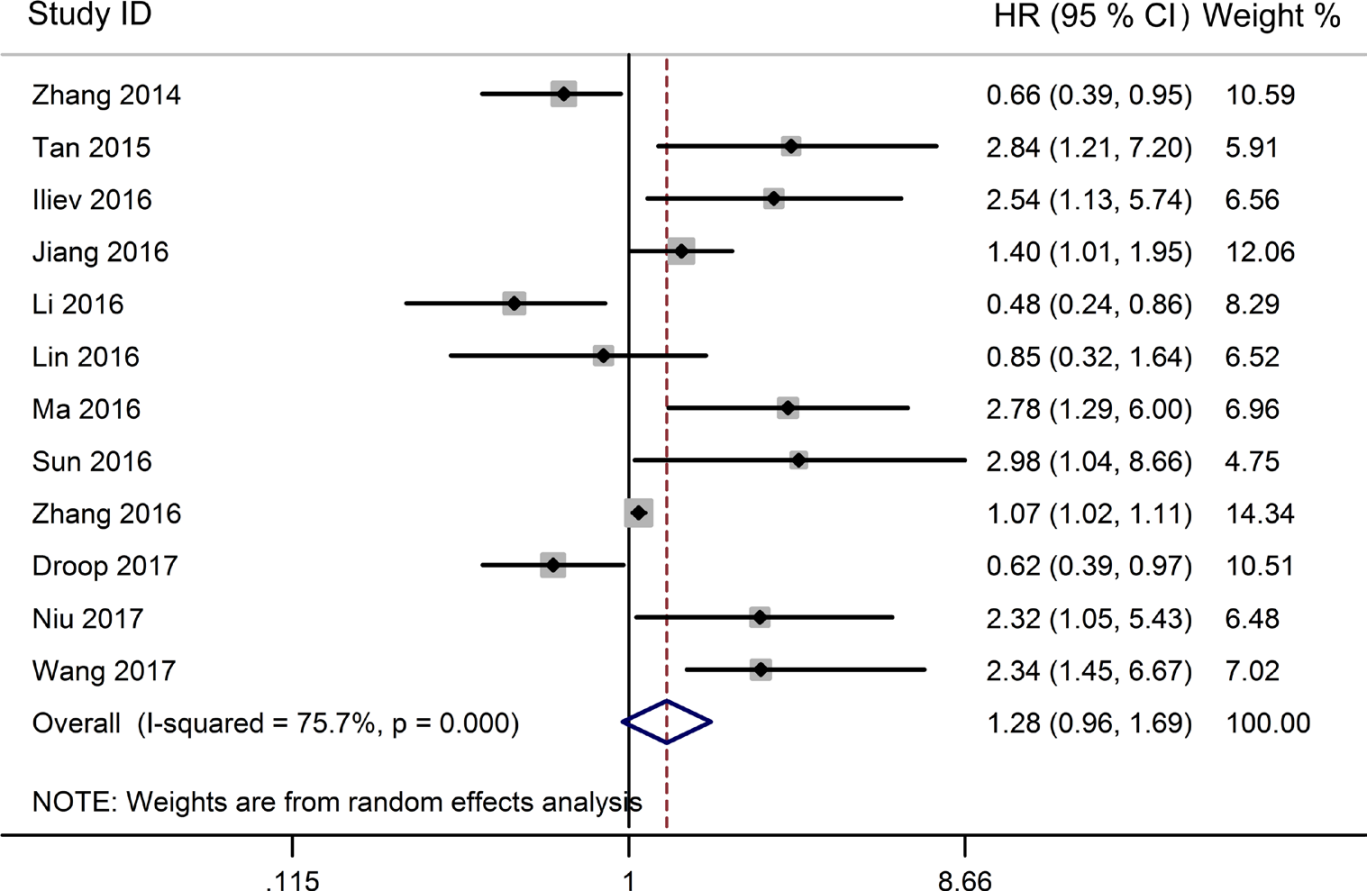
Forest plot for the relationship between TUG1 expression levels with OS

The association between TUG1 expression and overall survival (OS) was detected in 12 studies including 1358 patients (Table 1). Due to significant heterogeneity among studies (I^2^ = 75.7%, *P* < 0.001) was observed, the random-effects model was used to pool the results. The merged HR indicated no significant relationship between TUG1 expression and OS (HR = 1.28, 95% CI: 0.96–1.69, *P =* 0.091; random-effects model) (Figure 2). To minimize heterogeneity among OS datasets, we performed subgroup analyses according to cancer type, region, sample size, analysis method, and expression level. As the results showed in Table 2, the region subgroup and analysis method subgroup exhibited no association with OS, and significant heterogeneity were present. When sorting by cancer type, over-expression of TUG1 had an unfavorable prognostic value for bladder cancer (HR = 2.67, 95% CI: 1.47–4.87, *P* = 0.001) but no significant association with other tumors. When stratifying by sample size, high TUG1 expression was significantly related to poor OS in patients sample size less than 100 subgroup (HR = 2.08, 95% CI: 1.44–3.00, *P* < 0.001 with less heterogeneity), while the sample size more than 100 subgroup exhibited no correlation (HR=1.00, 95% CI: 0.743–1.37, *P* = 0.991). When grouped according to the expression level of TUG1 in cancer patients, the pooled HRs for the increased TUG1 expression subgroup and decreased TUG1 expression subgroup were 1.91(95% CI: 1.33–2.75, *P* < 0.001)) and 0.63 (95% CI: 0.48–0.82, *P* = 0.001 with less heterogeneity), respectively.

**Table 1:** Main characteristic of the eligible studies for meta-analysis

Study	Region	Tumor type	Sample size	Test method	Cut-off	Outcome measure	High TUG1 expression		Low TUG1 expression		Follow-up (months)
							LNM (Yes/No)	DM (Yes/No)	LNM (Yes/No)	DM (Yes/No)	
Zhang 2014	China	NSCLC	192	qRT-PCR	median value	OS	NA	NA	NA	NA	∼60
Tan 2015	China	BC	54	qRT-PCR	NA	OS	NA	NA	NA	NA	Over 50
Iliev 2016	Czech	MIBC	47	qRT-PCR	median value	OS	NA	NA	NA	NA	Over 100
Jiang 2016	China	ESCC	218	qPCR	NA	OS	86/22	NA	82/27	NA	Over 60
Kuang 2016	China	OC	62	qPCR	NA	NA	18/15	NA	12/17	NA	NA
Li 2016	China	Glioma	120	qRT-PCR	mean value	OS	NA	NA	NA	NA	∼60
Lin 2016	China	NSCLC	89	qRT-PCR	NA	OS	NA	NA	NA	NA	∼60
Li T 2016	China	BRC	100	qRT-PCR	mean value	NA	29/26	34/21	25/20	8/37	NA
Ma 2016	China	OSA	76	qRT-PCR	mean value	OS	NA	NA	NA	NA	∼60
Sun 2016	China	CRC	120	qRT-PCR	Five fold	OS	35/23	18/25	15/47	7/70	∼60
Zhang 2016	China	GC	100	qRT-PCR	median value	OS	30/20	3/47	27/23	2/48	∼60
Zhang M 2016	China	ccRCC	40	qRT-PCR	Two fold	NA	3/28	NA	1/8	NA	NA
Droop 2017	Germany	UC	106	qRT-PCR	median value	OS	NA	NA	NA	NA	∼200
Niu 2017	China	SCLC	33	qRT-PCR	NA	OS	NA	NA	NA	NA	Over 30
Wang 2017	China	ccRCC	203	qRT-PCR	NA	OS	29/71	32/68	13/90	15/88	Over 60

ESCC: esophageal squamous cell carcinoma; GC: gastric cancer; SCLC: small cell lung cancer; MIBC: muscle-invasive bladder cancer; NSCLC: non-small cell lung cancer; ccRCC: clear-cell renal cell carcinoma; CRC: colorectal cancer; OSA: osteosarcoma; UC: urothelial carcinoma; BC: bladder cancer; OC: ovarian cancer; BRC: breast cancer; OS: overall survival.

**Table 2:** Main results of subgroup analyses

Categories	Subgroups	Studies (*n*)	HR (95% CI)	*P*	Heterogeneity	
					*I*^2^ (%)	*P*_h_
All		12	1.28 (0.96, 1.69)	0.091	75.7	0.000
Cancer type	1)Digestive system cancers	3	1.29 (0.92,1.81)	0.144	68.0	0.044
	Respiratory system cancers	3	1.04 (0.49, 2.17)	0.927	71.3	0.031
	Urinary system cancers	4	1.71 (0.72, 4.09)	0.225	83.5	0.000
	Others	2	1.14 (0.20,6.37)	0.882	91.6	0.001
	2)NSCLC	2	0.70 (0.47,1.03)	0.073	0.0	0.594
	Bladder cancer	2	2.67(1.47, 4.87)	**0.001**	0.0	0.856
	Others	8	1.28(0.92, 1.80)	0.147	77.7	0.000
Region	Asia	10	1.32 (0.97, 1.79)	0.074	74.4	0.000
	Europe	2	1.20 (0.30, 4.80)	0.797	88.8	0.003
Sample size	≥ 100	7	1.00 (0.743 1.37)	0.991	77.4	0.000
	< 100	5	2.08 (1.44, 3.00)	**< 0.001**	32.5	0.205
Analysis method	Multivariate	6	1.38 (0.97, 1.96)	0.074	78.0	0.000
	Survival curves	6	1.23 (0.65, 2.30)	0.526	77.1	0.001
Expression level	Increased in tumors	8	1.91 (1.33, 2.75)	**< 0.001**	75.1	0.000
	Decreased in tumors	4	0.63(0.48, 0.82)	**0.001**	0.0	0.738

### Correlation of TUG1 expression with clinicopathological features

**Figure 3:**
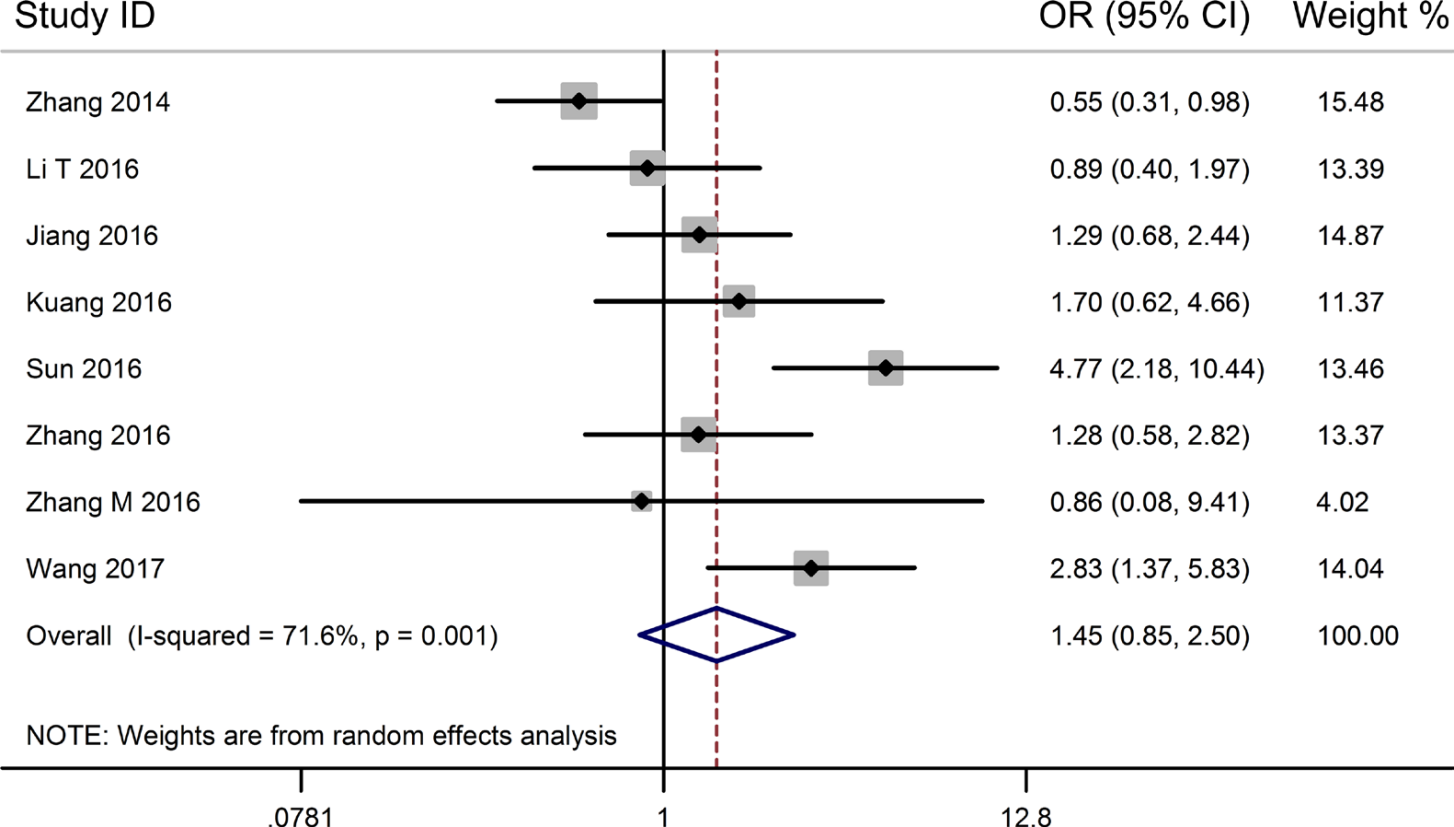
Forest plot for the relationship between TUG1 expression levels with LNM

**Figure 4:**
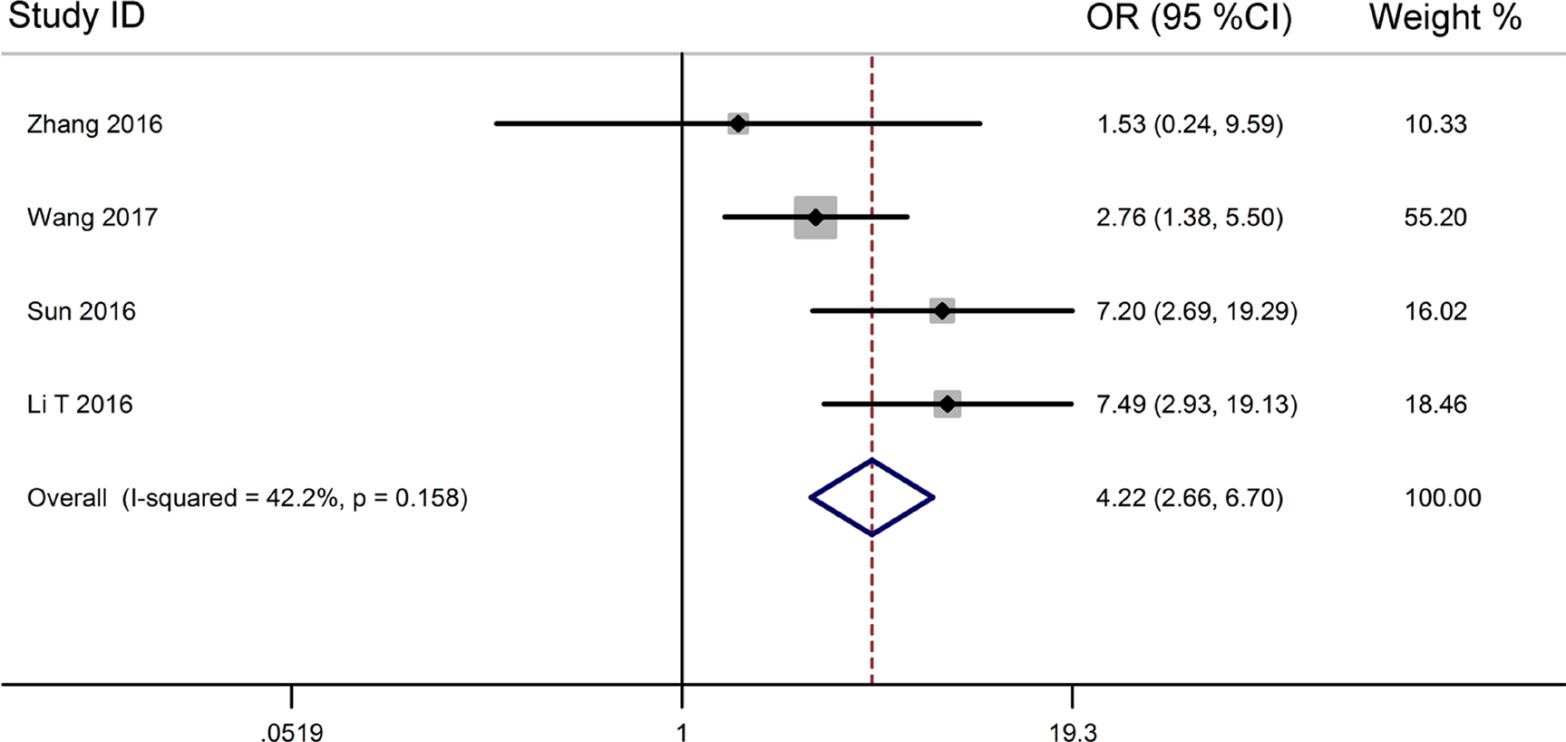
Forest plot for the relationship between TUG1 expression levels with DM

From the pooled results (Figure 3, Table 3), it found that high TUG1 expression was significantly associated with distant metastasis (DM) (OR = 4.22, 95% CI: 2.66–6.70, *P* < 0.001) (Figure 4, Table 3), and tumor differentiation (OR = 2.45, 95% CI: 1.28–4.70, *P* = 0.007) (Table 3). However, there was no significant correlation between the high TUG1 levels and gender (OR = 1.04, 95% CI: 0.77–1.42, *P* = 0.774) or age (OR = 0.75, 95% CI: 0.51–1.10, *P* = 0.136) or lymph node metastasis (OR = 1.45, 95% CI: 0.85–2.50, *P* = 0.177) or clinical TNM stage (OR = 0.55, 95% CI: 0.17–1.81, *P* = 0.326) (Table 3). Due perhaps to the inadequate data, we were failed to detect the association between the increased TUG1 expression and some other clinicopathological factors.

**Table 3:** Meta-analysis results of the associations of increased TUG1 expression with clinicopathological parameters

Clinicopathological parameter	Patients size	OR (95% CI)	*P* value	Heterogeneity	
				*I*^2^ (%)	*P*_h_
Gender(Male vs. Female)	952	1.00 (0.76–1.31)	0.978	0.0	0.536
Age ( Age > 60 vs. ≤ 60)	422	0.75 (0.51–1.10)	0.136	0.0	0.460
TNM stage( I/II vs. III/IV)	602	0.55 (0.17–1.81)	0.326	91.2	0.000
Differentiation ( Low /Undiffe vs. Middle/High)	641	2.45 (1.28–4.70)	**0.007**	70.0	0.019
Lymph node metastasis (Yes vs. No)	1034	1.45 (0.85–2.50)	0.177	71.6	0.001
Distant metastasis (Yes vs. No)	523	4.22 (2.66–6.70)	**< 0.001**	42.2	0.158

### Sensitivity analysis

**Figure 5:**
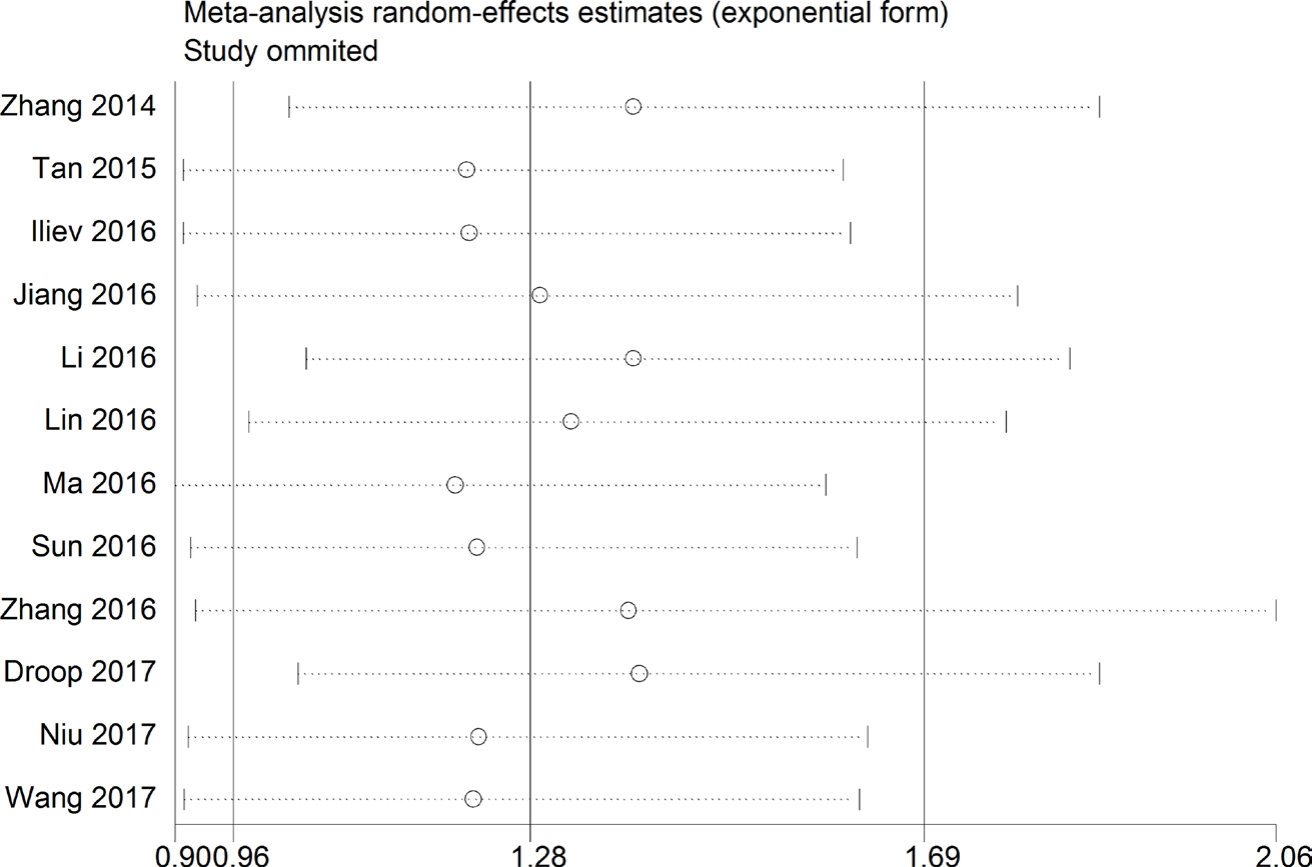
The sensibility analysis for this meta-analysis

A sensitivity analysis was conducted to evaluate the robustness of the summarized results, which were not significantly influenced by successively excluding each individual study from the pooled analysis. This suggested that the pooled HR of OS was robust (Figure 5).

### Publication bias

**Figure 6:**
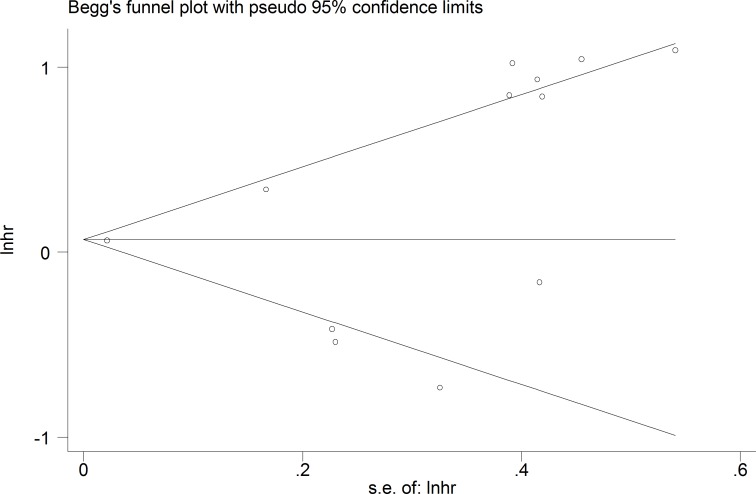
Funnel plot analysis of potential publication bias for meta-analysis

The publication bias of this meta-analysis was assessed by Begg’s funnel plot analysis. The funnel plot for the OS was asymmetric, suggesting potential publication bias (Figure 6). However, the Begg’s test showed no severe publication bias among the included studies (*Pr* > |z| = 0.150).

## DISCUSSION

Malignant neoplasm is one of the prevalent and deadly diseases worldwide. Recent years, accumulating evidences reveal that aberrantly expression of lncRNAs has been linked to tumorigenesis and neoplasm progression [6, 7, 21–23]. Across all cancer-related lncRNAs, TUG1 was a newly identified non-protein coding RNA gene, it participates in regulating proliferation and apoptosis in a variety of tumor cells [5–9, 24]. Interestingly, previous studies have shown that TUG1 were up-regulated in BRC [7], CRC [8], OC [9], SCLC [10], OSA [18], GC [19], ESCC [16], ccRCC [6, 20], BC [15] and MIBC [17], and function as an oncogene, while TUG1 were down-regulated in NSCLC [11, 12], glioma [13] as well as UC [14], and function as a tumor suppressor. Due to inconsistent evidence existed about the role of TUG1 in tumorigenesis and neoplasm progression was inconsistent. It is necessary to systematically explore the relationship between TUG1 expression and cancer.

To analyze the results of previous studies evaluating the relationship of TUG1 expression with cancer prognosis, we performed this comprehensive meta-analysis. A total of 15 eligible studies, comprising 13 common cancer types, met the selection criterions. The data of each study were handled according to OS, gender, differentiation, clinical stage, LNM and DM. The results of this meta-analysis indicated that no significant association between high TUG1 expression and OS of cancers (HR = 1.28, 95% CI: 0.96–1.69, *P =* 0.091), which was consistent with similar research of Yu et al. [25]. We also found increased TUG1 expression may be an unfavorable prognostic factor for bladder cancer based on subgroup analysis, which was consistent with similar research of Liu et al. [26]. In addition, high TUG1 expression is significantly correlated with DM (OR = 4.22, 95% CI: 2.66–6.70, *P* < 0.001) and tumor differentiation (OR=2.45, 95% CI: 1.28–4.70, *P* = 0.007). Furthermore, no significant correlations were observed between the high TUG1 expression and gender (OR = 1.04, 95% CI: 0.77–1.42, *P* = 0.774 or age (OR = 0.75, 95% CI: 0.51–1.10, *P* = 0.136) or lymph node metastasis (OR=1.45, 95% CI: 0.85–2.50, *P* = 0.177) or clinical TNM stage (OR = 0.55, 95% CI: 0.17–1.81, *P* = 0.326). However, the result of high TUG1 expression and LNM in our study was consistent with the result of Liu et al. [26] and contrary to Yang et al. [27]. To explore whether the heterogeneity affect the pooled results, we found increased TUG1 expression was positively associated with LNM (OR = 1.75, 95% CI: 1.09–2.81, *P* < 0.001, random effects model) and advanced clinical stage (OR = 0.33, 95% CI: 0.14–0.77, *P* = 0.013) after exclude a datasets from Zhang et al. [11]. Nevertheless, due to the small size of the study, this conclusion should be further verified.

There are some limitations of the current study that should be considered in explaining the results of this meta-analysis. Firstly, there was statistical heterogeneity in our present study. The sources of heterogeneity were diverse, such as cancer type, sample size, the region of patients, tumor stage, cut off value, and so on. Noteworthy, the expression pattern of TUG1 were inconsistent in tumors, TUG1 were up-regulated compared to adjacent tissues in some tumors [6–10, 15–20], while TUG1 were down-regulated in some other tumors [11–14]. We further analysis found that high TUG1 level were positively associated with poor OS in increased TUG1 expression subgroup (HR = 1.91, 95%CI: 1.33–2.75, *P* < 0.001), while increased TUG1 expression act as a favorable factor for OS in decreased TUG1 expression subgroup (HR = 0.63, 95%CI: 0.48–0.82, *P* = 0.001). Secondly, most of the included tumor patients from Chinese sample populations. Our results should be cautiously extended to other ethnic groups. Thirdly, some HRs and their corresponding 95%CIs were extracted from Kaplan-Meier curves, which are less reliable than those directly obtained from the primary studies. Finally, the cut-off values of increased TUG1 expression not consistent may be restricted to expand the clinical applicability.

In summary, our meta-analysis provides evidence that increased TUG1 expression is associated with poorer survival in cancer patients with high TUG1 expression but better survival in patients with low TUG1 expression. However, the results need to be confirmed by future studies with well-designed and larger-size in various tumors.

## MATERIALS AND METHODS

### Literature search strategy

Literature about lncRNA TUG1 expression was searched in the online electronic databases PubMed, Embase, Web of Science and CNKI (up to April 25, 2017). Search keywords or their combinations were as follows: “taurine upregulated gene 1 OR TUG1” AND “cancer OR tumor OR tumour OR neoplasm OR neoplasma OR neoplasia OR carcinoma OR glioma OR angiosarcoma OR lymphoma OR melanoma OR leukemia”. Only include English or Chinese articles in this study.

### Study selection criteria

The inclusion criteria for present study were as follows: (1) studies researched the association between TUG1 expression and prognosis of cancer patients; (2) the expression levels of TUG1 were divided into two groups: high or low; (3) complete data were available for computation of odds ratio (OR) or hazard ratio (HR) with 95% confidence interval (CI), and Kaplan-Meier curves or, if unavailable, related data obtained by contacting the corresponding authors.

The exclusion criteria for our meta-analysis include the following: (1) duplicate articles; (2) letters, case reports, expert opinions, editorials and reviews; (3) studies without available data; (4) sample cases fewer than 30; (5) non-human research.

### Date extraction and quality assessment

Two investigators (Na Li and Wei Li) independently using the Newcastle-Ottawa Quality Assessment Scale (NOS) to assess the quality of each include study, which were reported in previously studies [28, 29]. All included studies were considered to be of high quality based on NOS. The data from each included study were extracted and reviewed by two authors (Wei Li and Ke Shi) independently. To resolve the disagreements, a consensus was reached by another researcher (Na Li). The collected data were as follows: first author’s name, publication date, study region, tumor type, tumor stage, detection method of TUG1 expression, assessment criteria for TUG1 expression, sample size, total patients number, number of patients in the high and low TUG1 level group, number of patients with LNM and DM in each group, survival data analysis, follow-up period, HR and corresponding 95% CI. If the survival data not showed in articles directly, a request was made to the corresponding authors, or using the Engauge Digitizer v.4.1 software to obtain it from the Kaplan-Meier curves as previously described [29].

### Statistical methods

The STATA 12.0 software (Stata, College Station, Texas) was used to carry out all statistical analyses. Heterogeneity of pooled HRs or Ors among the eligible studies was judged by using the I-squared statistic; with I^2^ values > 50% indicating that significant heterogeneity was present. A fixed effects model was used to analyze the pooled results when the included studies without significant heterogeneity (I^2^ < 50 %). On the contrary, a random effects model was employed (I^2^ > 50%). Sensibility analysis was executed to evaluate the robustness of the overall results. Begg’s funnel plot was used to assess potential publication bias. Subgroup analysis was utilized to investigate the origin of heterogeneity. All the *P-*values less than 0.05 were regarded as statistical significance.
